# The Alarming Proximity of Parasites

**DOI:** 10.1371/journal.pbio.1000526

**Published:** 2010-11-02

**Authors:** Frédéric Thomas

**Affiliations:** IRD, MIVEGEC (UMR CNRS/IRD/UM), Team “Strategy and Adaptation of Transmission”, 911 Avenue Agropolis, 34090 Montpellier, France

**Figure pbio-1000526-g001:**
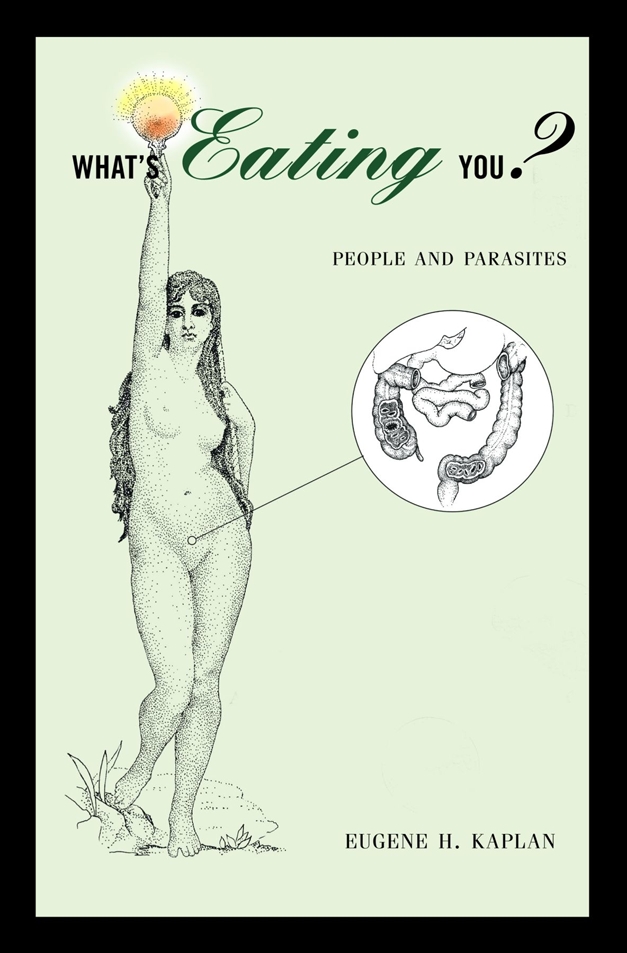
Kaplan EH (2010) What's Eating You? Princeton, NJ: Princeton University Press. 320p. ISBN 978-0-691-14140-4 (hardback). US$26.95.

[Fig pbio-1000526-g001]If you were to pick up and dissect any living organism from anywhere around the globe, you would undoubtedly find at least one other species inside of it—a symbiont, often a parasite. This ubiquity makes it clear that the way these passengers interact with their living habitats is key to understanding many aspects of life, primarily, the health and the well-being of free-living host species [1]. Like most animals, humans are replete with a large variety of symbiotic species, including parasites, which have plagued them since their origins and across their evolutionary history. No less than 179 species of parasites (eukaryotes) have been shown to parasitize *Homo sapiens sapiens*. Most of these can also parasitize other animals, but 35 of these infect only humans [2].

The vast majority of people are disgusted by parasites for obvious reasons. The idea that something is *eating you* is rather unpleasant. Beyond the purely psychological aversion, most infections are accompanied by a cortege of effects detrimental to your health. Some effects, like itchiness around the anus, are minor and disappear with the parasite on treatment, while others, from tuberculosis to elephantiasis, can cause great suffering and even death. Fully aware of such possible fates, people the world over naturally wonder about the parasites themselves: how do they cause infection? Are there risky behaviours that increase my chances of acquiring an infection? Which parasites do I need to be aware of whilst on holiday? What are my chances of recovery once infected and how a long will recovery take? Plenty of books, pamphlets, and websites address such questions, but Eugene Kaplan's book *What's Eating You?*, published earlier this year, gives us a unique and fresh perspective. And, as the title suggests, it's fun to read.

Kaplan is obviously fascinated by the world of parasites. Having spent much of his adult life travelling, encountering a wide variety of ecosystems, he has crossed paths with many different parasite species, and on more than one occasion has acquired infections himself! *What's Eating You?* is an easy-to-read book in which Kaplan recounts several of his adventures with parasites, titillating the curiosity of the reader with their “gross” vital statistics and often with subtle humour. Following a brief introduction defining symbiotic relationships (mutualism, commensalism, and parasitism), the book proceeds through a succession of 30 exciting true stories each with a focus on a different parasite, many devoted to human parasites. Kaplan also addresses extraordinary phenomena that capture the imagination, such as when parasites manipulate host behaviour leading to host suicide, or when brood parasites like cuckoos, force magpies to raise their chicks through a mafia-like strategy. Kaplan provides us with an elegant work combining instances of everyday life with a rich understanding of the natural history of symbiotic relationships that surround us or work within us.

Even those with “First World” lifestyles who consider parasites a distant problem, the book provides a reality check of their proximity plus many “tasty” and amusing anecdotes for conversations around (or best kept from) the dinner table. Among them we learn how Kaplan discovered that even men could give birth when he released an ascaris worm in the toilet. For those who already have a solid framework of knowledge of parasites, I believe that his informal approach will be well appreciated. Overall, I enjoyed this book considerably. For me it provides an excellent and comprehensive background that is both erudite and easily digestible.

For parasitologists, this book contains little new data per se, but the information concerning the biology of symbiotic organisms and their interactions with their hosts is relevant and accurate. Kaplan not only brings to view the importance and ubiquity of the parasitic world to a general audience, his text clearly invites curious readers to further explore each host–parasite relationship presented. Throughout he integrates evolutionary principles with those of parasitology, ecology, epidemiology, and behavioural ecology. The book contains neither photographs nor diagrams but is beautifully illustrated with drawings.

And so, to whom would I refer this book? To anyone, from students to travellers, as long as they have a passing interest in learning about the parasites that surround us, especially those that infect humans. This book is well adapted for an introductory parasitology course, as it presents a large spectrum of the most popular parasites (*Leishmania*, *Trypanosoma*, *Plasmodium*, *Ascaris*, *Onchocerca*, *Anisakis*, *Schistosoma*, etc.) and is easy to follow without specialized knowledge. It will serve as a useful anecdotal resource for teachers. In this context, however, the very short list of selected references provided is somewhat frustrating and certain references are very old and likely difficult to access.

The greatest strength of this book lies in its personal touch—it is a first-hand account of Kaplan's experience in the field. As biology students know, most textbooks on the biology of parasites are somewhat dry because they are encyclopaedically descriptive and emotionless. With Kaplan, the reader is being transported through real life experiences, evoking our morbid fascination with matters wormy and faecal. As expressed by Kaplan, “reality is the foundation of learning and students are currently starved for reality.” I share this point of view. Through all manner of disgusting and even frightening details, Kaplan makes attractive and easy to follow what is usually soporific in other books.
